# Estimated number of reported vaccine-preventable disease cases averted following the introduction of routine vaccination programs in Sweden, 1910–2019

**DOI:** 10.1093/eurpub/ckad169

**Published:** 2023-10-26

**Authors:** Leah J Martin, Ilias Galanis, Tiia Lepp, Ann Lindstrand

**Affiliations:** Public Health Agency of Sweden, Solna, Sweden; Public Health Agency of Sweden, Solna, Sweden; Public Health Agency of Sweden, Solna, Sweden; Public Health Agency of Sweden, Solna, Sweden

## Abstract

**Background:**

Routine childhood vaccination programs have had enormous positive public health impacts worldwide. However, in some areas, these benefits may be impeded by vaccine hesitancy and undervaccination. We estimated the number of reported cases of measles, pertussis, mumps and poliomyelitis averted in Sweden after the introduction of routine childhood vaccination programs.

**Methods:**

We used annual national data on population size and the number of reported cases of measles (1911–2019), pertussis (1911–2019), mumps (1914–2019) and poliomyelitis (1910–2019) for Sweden. For each disease, we calculated the median and 95% confidence interval of the annual pre-vaccination incidence to estimate the number of counterfactual cases; that is, the estimated number of cases that would have been observed in the post-vaccination period had no vaccine been introduced (median incidence × average annual population). For the post-vaccination periods, we calculated reported cases averted and assumed all decreases were due to vaccines.

**Results:**

In total, for all four diseases combined, over 2.1 million cases were reported over the respective surveillance periods. Since the introduction of vaccinations, we estimate that over 1.5 million reported cases of these four diseases combined have been averted: measles (633 091), pertussis (608 670), mumps (262 951) and poliomyelitis (58 240). However, due to underreporting, especially during pre-vaccination years, these are likely underestimates.

**Conclusions:**

Since the introduction of these routine childhood vaccination programs in Sweden, a substantial number of reported cases of vaccine-preventable diseases have been averted. Vigilance against both failure to vaccinate and undervaccination is necessary to prevent future increases of these vaccine-preventable diseases.

## Introduction

Routine childhood vaccination programs have had enormous positive public health impacts worldwide. In Sweden, vaccination is voluntary and immunization coverage has been, and remains, high.^1^^,^^2^ However, this high coverage can create complacency and reduce awareness about the severity of vaccine-preventable diseases (VPDs) and the historic burden of these diseases in society. In some sub-populations and areas of the country, the public health benefits of vaccines may be impeded by undervaccination and vaccine hesitancy,^3^ which can be defined as ‘delay in acceptance or refusal of vaccination despite availability of vaccination services’.^4^

The resurgence of measles in many countries in 2018–19 was an example of the effects of sub-optimal vaccination coverage and clusters of unvaccinated individuals, with an increased number of cases and outbreaks reported in many countries worldwide including the USA^5^ and in Europe.^6^ In 2019, measles transmission was considered to be re-established in six European countries (Albania, the Czech Republic, Lithuania, Slovakia, the UK and Uzbekistan).^7^

In response to vaccine hesitancy and the increased number of cases of VPDs, several countries, including the USA,^8^ The Netherlands^9^ and Italy,^10^ have estimated the number of reported cases of VPDs averted since the introduction of routine vaccination programs. These results illustrate the historic impact of vaccination and help serve as a baseline for the future. However, to our knowledge, these estimates are not yet available for any of the Nordic countries. Therefore, our aim is to estimate the number of reported VPD cases averted nationally in Sweden after the introduction of childhood vaccination programs.

## Methods

### Data

This is a population-based study using national population data for Sweden^11^ and annual, national reported surveillance data for four VPDs: measles (1911–2019, vaccine introduced 1971), pertussis (1911–2019, vaccine introduced 1953), poliomyelitis (1910–2019, vaccine introduced 1957) and mumps (1914–2019, vaccine introduced 1982). These four VPDs are currently notifiable diseases,^12^ with case-based reporting by both clinicians and laboratories; however, surveillance has varied by disease and time, which affects the reporting of cases. For example, surveillance data for mumps were unavailable from 1954 to 1968. Ethics approval was not needed for this study as we used data collected for public health surveillance purposes.

In Sweden, vaccination is voluntary. Details of vaccination program changes are shown in table 1. Notably, a whole cell vaccine for pertussis was introduced in 1953 and withdrawn in 1979 which began a 17-year period in which vaccination against the disease was not provided by any vaccination program until 1996 when an acellular pertussis vaccine was introduced. Other changes occurred in the vaccination schedule over time but we did not specifically consider these in this analysis.^13^

**Table 1:** Vaccine-preventable diseases in Sweden: number of reported cases and incidence by vaccination program status

Disease	Vaccine program status	Period	Duration of period (years)	Median annual incidence per 100 000 population (95% CI)^a^	No. of cases reported
Pertussis	Pre-vaccination	1911–52	42	166.0 (146.3–191.2)	437 862
	Whole cell vaccine	1953–78	26	72.9	173 670
	Vaccine withdrawn	1979–95	17	84.4	132 373
	Acellular vaccine	1996–2019	24	7.6	30 692
		Total			774 597

Poliomyelitis	Pre-vaccination	1910–56	47	10.9 (7.1–14.1)	52 375
	Vaccine	1957–2019	63	0.0	667
		Total			53 042

Measles	Pre-vaccination	1911–70	60	173.2 (113.9–245.1)	815 470
	Measles vaccine	1971–81	11	104.5	104 167
	MMR vaccine	1982–2019	38	0.3	13 370
		Total			933 007

Mumps^b^	Pre-vaccination	1914–53	40	65.3	218 049
		1954–68 (no surveillance)	15	–	–
		1969–81	13	117.8	164 099
		Total pre-vaccination period	68^b^	85.3 (58.5–104.1)	382 148
	MMR vaccine	1982–2019	38	0.4	29 531
		Total			411 679

Overall total					2 172 325

Note: MMR, measles, mumps and rubella.

a 95% confidence interval (CI) estimated for the pre-vaccination period.

b For mumps, no surveillance was conducted during 1954–68 (15 years). Analyses were based on the 53 pre-vaccination years with reported case data.

### Analysis

Our analysis is based, in part, on previous work by van Panhuis *et al*.^8^ For each disease, annual incidence was calculated as the number of reported cases for the year divided by the average population (the average of the end-of-year population for that year and the previous year). We estimated the median annual pre-vaccination incidence for each disease and, by creating 100 000 non-parametric bootstrap samples, estimated the upper and lower 95% confidence limits of this median using the BCa method.^14^ We used this median to estimate the annual number of counterfactual cases (median incidence × average annual population), which we summed over the relevant post-vaccination period to estimate the number of cases that would have been observed had no vaccine been introduced. Similarly, using the median, we estimated averted cases (counterfactual cases − observed cases) and percent averted cases (averted cases/counterfactual cases × 100). We assumed that all decreases in VPD incidence in the post-vaccination periods were due to vaccines.

We considered the pre-vaccination period to end the year before the introduction of the first vaccine against the disease. In our main analysis, we used the full pre-vaccination periods for each disease. In addition, as a sensitivity analysis, we used the pre-vaccination period beginning in 1945 to examine the likely effects of underreporting in the earlier years on our results; this was meant to approximate a post-World War II period, based on the work of others.^9^ We compared the percentage difference in estimated cases averted between the two analyses. However, for pertussis especially, and poliomyelitis, this resulted in a relatively lower number of data points for analysis. Additionally, we conducted both the main and sensitivity analyses using the mean incidence (Supplementary material). Analyses were conducted in R version 4.1.3.^15^

## Results

**Figure 1:**
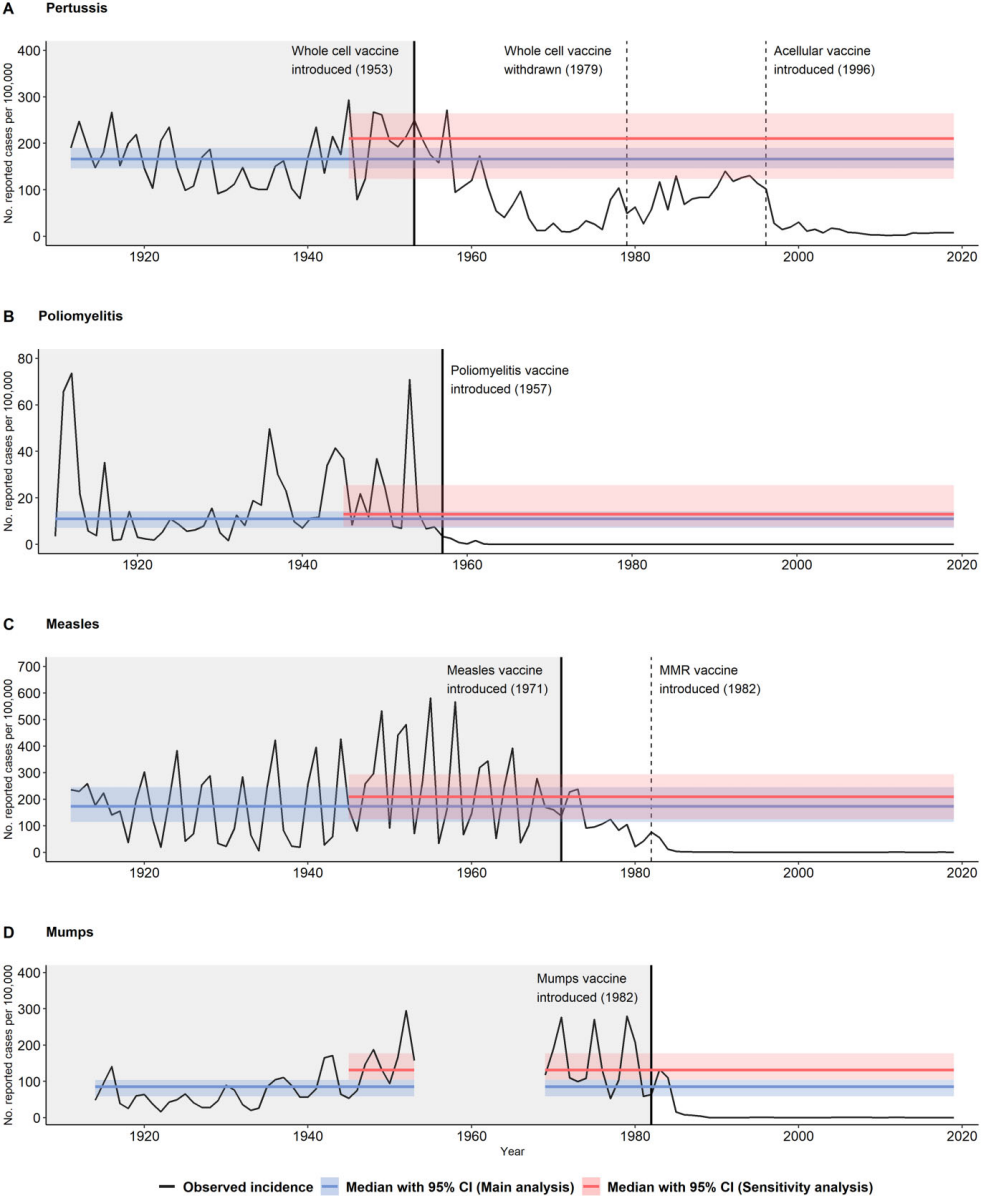
Number of reported vaccine-preventable disease cases per 100 000 population: observed incidence and median (95% CI) pre-vaccination incidence based on the main and sensitivity analyses, Sweden 1910–2019. Notes: Gray-shaded areas represent pre-vaccination periods. Scales on the *y*-axes are not consistent. CI, confidence interval

In total, for all four VPDs combined, 2 172 325 cases were reported over the respective surveillance periods, which varied greatly by disease, with the pre-vaccination median incidence per 100 000 ranging from 10.9 [95% confidence interval (CI): 7.1–14.1] for poliomyelitis to 173.2 (95% CI: 113.9–245.1) for measles (table 1, figure 1). Since the introduction of the routine vaccination programs considered, we estimate that a total of 1 562 952 reported cases of these four diseases combined have been averted (table 2). Similar to the number of reported cases, the estimated number of reported cases averted varied greatly by disease. The highest number of estimated reported cases averted was for measles (633 091) and pertussis (608 670) followed by mumps (262 951), while the lowest was for poliomyelitis (58 240) (table 2). The estimated percentage of reported cases averted ranged from 64.4% for pertussis to 98.9% for poliomyelitis; overall, 76.3% of reported cases were estimated to be averted (table 2).

**Table 2:** Estimated number of counterfactual and averted reported cases of vaccine-preventable diseases after introduction of vaccination programs, Sweden

Disease	Period	Estimated counterfactual cases	Estimated cases averted due to vaccination	Estimated % cases averted
Pertussis				
	1953–78	334 926	161 256	48.1
	1979–95	239 216	106 843	44.7
	1996–2019	371 262	340 570	91.7
	Total (1953–2019)	945 405	608 670	64.4

Poliomyelitis				
	Total (1957–2019)	58 907	58 240	98.9

Measles				
	1971–81	156 582	52 415	33.5
	1982–2019	594 045	580 675	97.7
	Total (1971–2019)	750 628	633 091	84.3

Mumps				
	Total (1982–2019)	292 482	262 951	89.9

Overall total		2 047 422	1 562 952	76.3

Note: Totals may not sum due to rounding.

In our sensitivity analyses, using the post-1945 pre-vaccination periods, we estimated a total of 2 137 367 reported cases averted, 36.8% higher than using the full pre-vaccine period (table 3). The largest percentage difference in the number of estimated reported cases averted was for mumps (59.8%, with 420 237 cases averted) followed by pertussis (41.2%, with 859 628 cases averted) (table 3).

**Table 3:** Sensitivity analysis: estimated number of counterfactual and averted reported cases of vaccine-preventable diseases based on data from 1945 and onwards until the introduction of the respective vaccination programs, Sweden

Disease	Pre-vaccination period (duration, years)	Median annual pre-vaccination incidence per 100 000 population (95% CI)	Post-vaccination period	Estimated counterfactual cases	Estimated cases averted due to vaccination	Difference in averted cases compared to main analysis	Percentage difference in averted cases relative to main analysis^a^	Estimated % cases averted
Pertussis	1945–52 (8)	210.0 (123.8–264.4)	1953–2019	1 196 363	859 628	250 958	41.2	71.9
Poliomyelitis	1945–56 (12)	12.9 (7.5–25.4)	1957–2019	69 939	69 272	11 031	18.9	99.0
Measles	1945–70 (26)	209.0 (124.5–293.4)	1971–2019	905 768	788 231	155 140	24.5	87.0
Mumps	1945–53, 1969–81 (22)	131.2 (103.9–177.2)	1982–2019	449 768	420 237	157 286	59.8	93.4

			Total	2 621 837	2 137 367	574 415	36.8	81.5

Note: CI, confidence interval, totals may not sum due to rounding.

a (Averted cases_Sensitivity analysis_ − Averted cases_Main analysis_)/Averted cases_Main analysis_ × 100.

## Discussion

Since the introduction of routine vaccination programs in Sweden, we estimate that a combined total of over 1.5 million reported cases of measles, mumps, pertussis and poliomyelitis have been averted. In addition to characteristics of the disease itself, differences in the number of reported cases averted for each disease can be explained by several factors, including variations in the surveillance methodology and reporting, pre-vaccination incidence rates, length of post-vaccination periods and effectiveness of the vaccines. Based on our analysis, the highest number of reported cases averted was for measles followed by pertussis, which had the highest and second highest median pre-vaccination incidence rates, respectively. During the post-vaccination periods considered in our study, the median observed incidence per 100 000 ranged from zero cases for poliomyelitis and near zero for measles and mumps (0.3 and 0.4 cases, respectively) and up to 7.6 cases for pertussis.

Pertussis has a unique history in Sweden, as the vaccine was withdrawn from routine vaccination programs from 1979 to 1995. This distinctive feature of the Swedish data, and the availability of surveillance data during this time, allows us to compare the counterfactual cases with the increase in reported case data. During the latter part of this 17-year withdrawal period, after the effects of herd immunity would have been reduced, the annual incidence peaked at 140.0 cases per 100 000 in 1991 (median = 84.4 cases per 100 000 during 1979–95). This peak nearly reached the lower 95% CI of the pre-vaccination median incidence in our main analysis (figure 1A), which suggests potential realism in our counterfactual scenario for pertussis. Nevertheless, even after the introduction of the acellular pertussis vaccination program in 1996, pertussis has continued to circulate in the community. The disease is of particular concern for infants who are at risk of severe illness if infected but are too young to be fully vaccinated.^16^

Other studies have also reported substantial reductions in the number of reported VPD cases in other countries. Pezzotti *et al*.^10^ estimated that over 1.7 million cases of the four diseases we studied were prevented in Italy over post-vaccination periods ranging from 17 to 52 years. In The Netherlands, van Wijhe *et al*.^9^ estimated the percentage of reported cases averted for poliomyelitis (93%) and mumps (79%) in the 13-year post-vaccination periods (less 9 months for mumps) immediately following introduction of each of the vaccines. These values are somewhat lower compared to our findings; however, we examined the entire post-vaccination period up to 2019, allowing for more averted cases to be observed. Additionally, Li *et al*.^17^ estimated that, in 98 low- and middle-income countries, vaccination against 10 diseases has or will avert 69 million deaths from 2000 to 2030. In this study, measles vaccination was associated with the largest number of averted deaths: 56 million (95% credible interval: 39–74 million).^17^

Comparisons between the data for Sweden and other countries must consider differences in many factors, including the duration of post-vaccination periods, immunization coverage, surveillance systems and the demographics and size of the populations. In Italy, for example, vaccination coverage for measles, mumps and rubella (MMR) at 24 months of age (in 2015) was just over 85%.^10^ In comparison, in Sweden, MMR immunization coverage among 2-year olds has been >95% for children born between 1989 and 2018 except for those born between 1998 and 2002, when it dropped to between 88.5% and 94.5%.^1^^,^^2^ For pertussis, the study from Italy reports a much lower pre-vaccination incidence rate than our study does for Sweden (42.79 vs. 166.0 cases per 100 000).^10^ However, in this Italian study, the pre-vaccine period includes years in which whole cell vaccine was recommended, though coverage remained low until 1995 when the acellular vaccine was introduced^10^; this could be one reason for the lower pre-vaccine incidence rates of pertussis reported for Italy. Furthermore, it is difficult to make direct comparisons based on the absolute number of reported cases averted; the percentage of reported cases averted is a more comparable metric, but this is not always available.

Estimating the counterfactual cases based on the pre-vaccination incidence presents a number of issues for consideration,^8^^,^^9^ including the duration of the pre-vaccination period to use as the baseline and choice of measure of central tendency (e.g. median or mean). As we saw in our sensitivity analysis, using the post-1945 pre-vaccination period as the baseline resulted in a higher median incidence compared to the main analysis for each disease. This difference is likely attributable, in large part, to higher underreporting rates in the earlier pre-vaccination years. This underreporting, in turn, likely led us to underestimate the number of averted cases, especially in the main analysis; this underestimation was likely greater for some diseases (e.g. mumps) than others (e.g. poliomyelitis). Finally, the choice of the median was made based on the positive skewness that the majority of the annual disease incidences presented in the pre-vaccination periods (Supplementary figure S1), thus resulting in more conservative counterfactual estimates compared with the mean.

Strengths of our study include our long surveillance period, with at least 109 years of data available for most of the diseases covered (except mumps), including 60 years of pre-vaccine data for measles, and the unique data on pertussis. In comparison, the Dutch study by van Wijhe *et al*.^9^ did not include measles or pertussis because no pre-vaccination data were available. In the US study, no pertussis data were available for 1955–74,^8^ a period during which our data are able to show the effect of using the whole-cell pertussis vaccine. The study from Italy also included data on pertussis, though it is difficult to compare, as mentioned above.

Our analysis has a number of limitations. First, underreporting occurred, especially during the pre-vaccination periods, which led to underestimates that we cannot completely account for. For example, in the pre-vaccination period, childhood infection with MMR was almost universal^18^; however, our numbers do not include all of these cases. On the other hand, paralytic poliomyelitis would have been less likely to be underreported. Surveillance varied by disease and evolved over time, which likely affected underreporting rates. As such, changes in observed incidence rates cannot be considered entirely attributable to real changes in the number of cases of disease. Therefore, our results likely underestimate the true number of cases averted and the likelihood of underestimation is likely to vary by disease. We have attempted to examine these differences in our sensitivity analysis. Second, we used national data aggregated annually and thus lacked finer spatial and temporal granularity that would be possible with more detailed data, which also reduced the number of data points available for the sensitivity analysis in particular. Monthly reported case data for these diseases are available in archived reports^19^; however, they are not available for all diseases each year and have not been digitized. Demographic data were not included for cases as they were only readily available for those occurring after 1996. Third, we did not incorporate immunization coverage rates in our analysis; however, it would be unlikely to change our overall results substantially because coverage has historically been, and continues to be, high in Sweden.^1^^,^^2^ Additionally, we did not know the vaccination status of the cases reported after the implementation of vaccination programs. Fourth, we did not consider severity of disease or mortality resulting from infection nor how this could have differed among the four diseases considered; these outcomes would have provided a more complete description of the impact of vaccination in Sweden. However, it is likely that the cases reported are more severely ill and vaccination has prevented serious illness and death. Fifth, our method assumes the absence of a trend in the pre-vaccination period. Although increases and decreases in true incidence occurred, these rates were also affected by improved surveillance and reporting over time. Therefore, we did not consider it appropriate to attribute changes in observed incidence rates entirely to real changes in the number of cases of disease. Finally, we assume that all decreases in VPD incidence in the post-vaccination periods were due to vaccines. However, other factors not considered in this analysis, such as socioeconomic status, living conditions, hygiene and sanitation, have improved since the introduction of the vaccination programs and may have contributed to reducing the burden of some of these diseases in society. This creates a degree of uncertainty in the number of cases averted as a result of vaccination programs. Nevertheless, as others note, the clear and abrupt decrease in the incidence of these diseases after the introduction of effective vaccination programs and their continued low incidence provide evidence for vaccination as a primary driver of disease prevention.^8^^,^^9^

In conclusion, since the introduction of routine vaccination programs in Sweden, a substantial number of cases of reported VPDs have been averted. Our results provide evidence to support the substantial, long-term public health impact of vaccination programs in Sweden over more than half a century. Vigilance against both undervaccination and failure to vaccinate with high and equitable coverage is necessary to prevent future increases of these VPDs.

## Supplementary Material

ckad169_Supplementary_Data

## Data Availability

The data underlying this article will be shared on reasonable request to the corresponding author.

## References

[1] Socialstyrelsen [The National Board of Health and Welfare]. Vaccination av barn: Det Svenska Vaccinationsprogrammet. [Vaccination of children: The Swedish Vaccination Programme]. Stockholm, Sweden: Socialstyrelsen; 2008. Article No. 2008-126-9.

[2] Folkhälsomyndigheten [The Public Health Agency of Sweden]. Barnvaccinationer efter vaccin, region och år 2002–2020. Andel (procent). [Childhood Vaccinations by Vaccine, Region and Year 2002–2020. Share (Percent)]. Available at: http://fohm-app.folkhalsomyndigheten.se/Folkhalsodata/pxweb/sv/A_Folkhalsodata/A_Folkhalsodata__L_Vaccin__BarnvacRegion/Barnvac.px/table/tableViewLayout1/ (30 May 2023, date last accessed).

[3] Folkhälsomyndigheten [The Public Health Agency of Sweden]. Barriers and Motivating Factors to MMR Vaccination in Communities with Low Coverage in Sweden. Stockholm, Sweden, 2015. Article No. 15027. Available at: https://www.folkhalsomyndigheten.se/contentassets/5db4b41a40f94e98b0e1d0d4a596bae8/barriers-motivating-factors-mmr-vaccination-communities-low-coverage-sweden-15027.pdf (5 June 2023, date last accessed).

[4] MacDonald NE ; SAGE Working Group on Vaccine Hesitancy. Vaccine hesitancy: definition, scope and determinants. Vaccine 2015;33:4161–4. 25896383 10.1016/j.vaccine.2015.04.036

[5] Patel M , Lee AD, Clemmons NS, et al National update on measles cases and outbreaks—United States, January 1–October 1, 2019. MMWR Morb Mortal Wkly Rep 2019;68:893–6. 31600181 10.15585/mmwr.mm6840e2 PMC6788396

[6] World Health Organization. Measles in the WHO European Region: Situation Report #3. 2019. Available at: https://www.euro.who.int/__data/assets/pdf_file/0020/420932/WHO-Measles-Sitrep-Dec-2019.pdf (19 October 2020, date last accessed).

[7] World Health Organization. Regional Office for Europe. 9th Meeting of the European Regional Verification Commission for Measles and Rubella Elimination (RVC). 2021. Report No. WHO/EURO:2021-4369-44132-62279. Available at: https://apps.who.int/iris/handle/10665/350211 (5 June 2023, date last accessed).

[8] van Panhuis WG , Grefenstette J, Jung SY, et al Contagious diseases in the United States from 1888 to the present. N Engl J Med 2013;369:2152–8. 24283231 10.1056/NEJMms1215400 PMC4175560

[9] van Wijhe M , Tulen AD, Korthals Altes H, et al Quantifying the impact of mass vaccination programmes on notified cases in the Netherlands. Epidemiol Infect 2018;146:716–22. 29534768 10.1017/S0950268818000481 PMC9134361

[10] Pezzotti P , Bellino S, Prestinaci F, et al The impact of immunization programs on 10 vaccine preventable diseases in Italy: 1900–2015. Vaccine 2018;36:1435–43. 29428176 10.1016/j.vaccine.2018.01.065

[11] Statistiska Centralbyrån [Statistics Sweden]. Population and Population Changes 1749–2019. Available at: http://www.scb.se/en/finding-statistics/statistics-by-subject-area/population/population-composition/population-statistics/pong/tables-and-graphs/yearly-statistics–the-whole-country/population-and-population-changes/ (19 September 2019, date last accessed).

[12] Folkhälsomyndigheten [The Public Health Agency of Sweden]. Notifiable Diseases. Available at: http://www.folkhalsomyndigheten.se/the-public-health-agency-of-sweden/communicable-disease-control/surveillance-of-communicable-diseases/notifiable-diseases/ (24 May 2021, date last accessed)].

[13] Folkhälsomyndigheten [The Public Health Agency of Sweden]. Tidigare vaccinationsprogram [Previous Vaccination Program]. Available at: http://www.folkhalsomyndigheten.se/smittskydd-beredskap/vaccinationer/vaccinationsprogram/tidigare-vaccinationsprogram/ (19 October 2020, date last accessed).

[14] DiCiccio TJ , Efron B. Bootstrap confidence intervals. Stat Sci 1996;11:189–228.

[15] R Core Team. R: A Language and Environment for Statistical Computing. Vienna, Austria: R Foundation for Statistical Computing; 2022. Available at: http://www.R-project.org.

[16] Nilsson L , Von Segebaden K, Blennow M, et al Kikhosta en risk för spädbarn. Sjukdomen cirkulerar bland ungdomar och vuxna. [Whooping cough is a risk to infants. The disease is circulating among adolescents and adults]. *Lakartidningen* 2013 Sep 11-17;110:1599–602. 24163932

[17] Li X , Mukandavire C, Cucunubá ZM, et al; Vaccine Impact Modelling Consortium. Estimating the health impact of vaccination against ten pathogens in 98 low-income and middle-income countries from 2000 to 2030: a modelling study. Lancet 2021;397:398–408. 33516338 10.1016/S0140-6736(20)32657-X PMC7846814

[18] Centers for Disease Control and Prevention. Measles, Mumps, and Rubella (MMR) Vaccination: What Everyone Should Know. Available at: https://www.cdc.gov/vaccines/vpd/mmr/public/index.html (31 May 2023, date last accessed).

[19] Statistiska Centralbyrån [Statistics Sweden]. Sök, Äldre statistik, SOS 1911- [Search – Older statistics, SOS 1911-]. Available at: https://www.scb.se/hitta-statistik/sok/Index?Subject=H%C3%A4lso-%20och%20sjukv%C3%A5rd&Series=SOS%201911-&From=1911&To=1996&Sort=relevance&Query=&Page=1&Tab=older&Exact=False (23 May 2023, date last accessed).

