# Supplementary material for: Estimated number of reported vaccine-preventable disease cases averted following the introduction of routine vaccination programs in Sweden, 1910–2019
Source: Eur J Public Health. 2023 Oct 26;33(6):1188–93. doi: 10.1093/eurpub/ckad169 (PMC10710358; doi:10.1093/eurpub/ckad169)
Supplement: ckad169_Supplementary_Data [file ckad169_supplementary_data.docx]

**Supplemental Materials**

**Title:** Estimated number of reported vaccine-preventable disease cases averted following the introduction of routine vaccination programs in Sweden, 1910-2019

**Authors:** Leah J Martin, Ilias Galanis, Tiia Lepp, Ann Lindstrand

**Affiliation:** Public Health Agency of Sweden, 171 82 Solna, Sweden

**Journal:** European Journal of Public Health


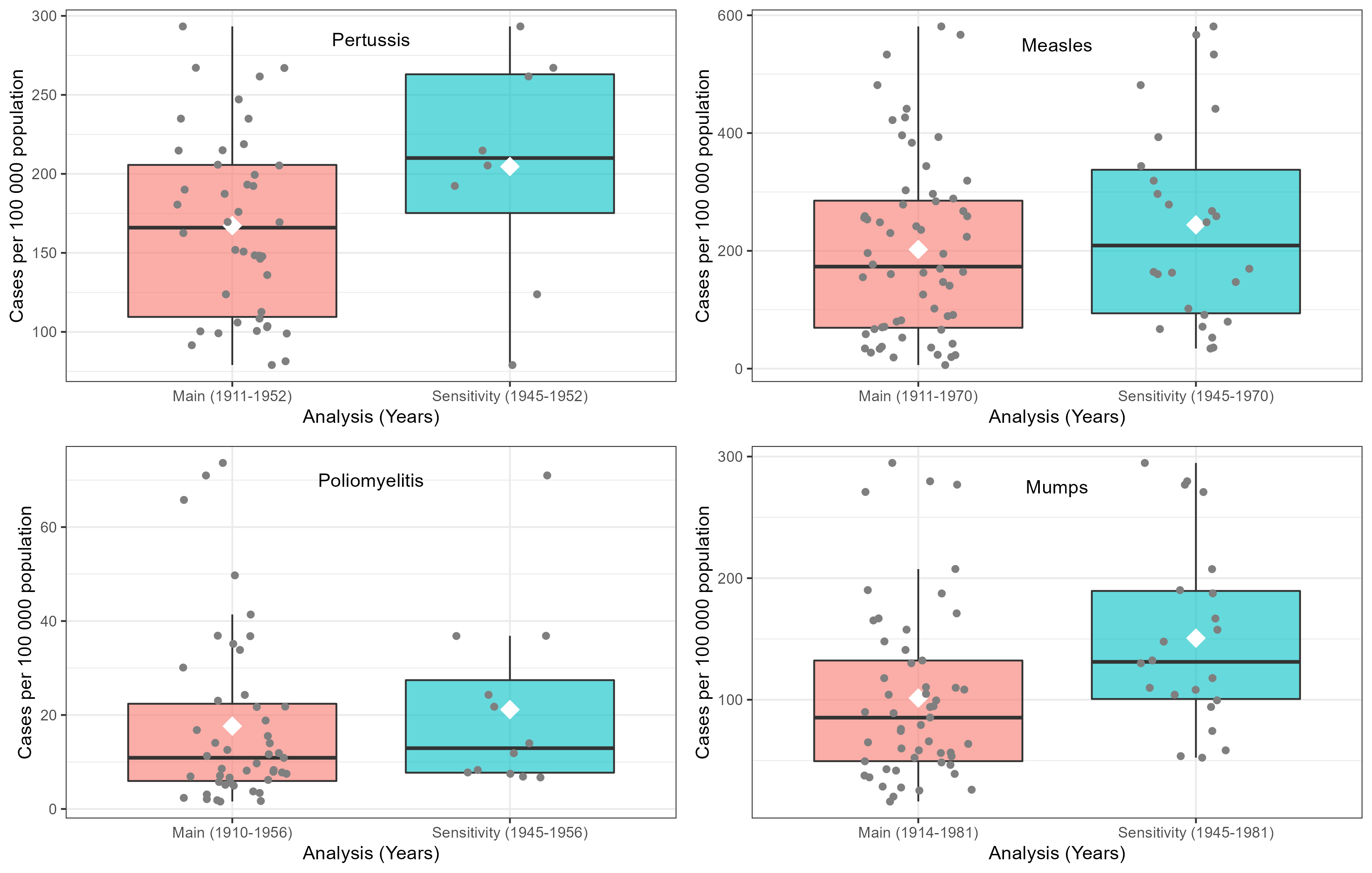


**Figure S1.** Reported cases per 100,000 population for each disease by pre-vaccination period: Main vs. sensitivity analysis.

Notes: Mumps is missing data for 1954-1968; scales differ by disease.

The thick black lines indicate the median, the lower and upper box edges indicate the 25^th^ and 75^th^ percentiles, respectively. Each gray dot indicates the annual incidence for one year, randomly placed along the x-axis. White diamonds indicate the mean for the pre-vaccination period considered.

**Table S1.** Mean and median incidence by disease and pre-vaccination period

| **Disease** | **Vaccination introduced** | **Analysis** | **Pre-vaccination period** | **Duration (years)** | **Annual incidence (per 100,000 population)** | |
| --- | --- | --- | --- | --- | --- | --- |
|  |  |  |  |  | **Median (95% CI)** | **Mean (95% CI)** |
| Pertussis | 1953 | Main | 1911-1952 | 42 | 166.0 (146.3-191.2) | 167.2 (150.6-185.1) |
|  |  | Sensitivity | 1945-1952 | 8 | 210.0 (123.8-264.4) | 204.7 (149.7-245.9) |
| Poliomyelitis | 1957 | Main | 1910-1956 | 47 | 10.9 (7.1-14.1) | 17.6 (13.3-23.9) |
|  |  | Sensitivity | 1945-1956 | 12 | 12.9 (7.5-25.4) | 21.1 (13.4-36.2) |
| Measles | 1971 | Main | 1911-1970 | 60 | 173.2 (113.9-245.1) | 202.3 (166.4-243.2) |
|  |  | Sensitivity | 1945-1970 | 26 | 209.0 (124.5-293.4) | 244.2 (184.7-314.5) |
| Mumps* | 1982 | Main | 1914-1953,  1969-1981 | 68* | 85.3 (58.5-104.1) | 101.4 (84.5-122.8) |
|  |  | Sensitivity | 1945-1953,  1969-1981 | 22 | 131.2 (103.9-177.2) | 150.7 (122.7-184.7) |

* For mumps, no surveillance was conducted during 1954-1968 (15 years). Analyses were based on the 53 pre-vaccination years with reported case data.

Note: CI = confidence interval


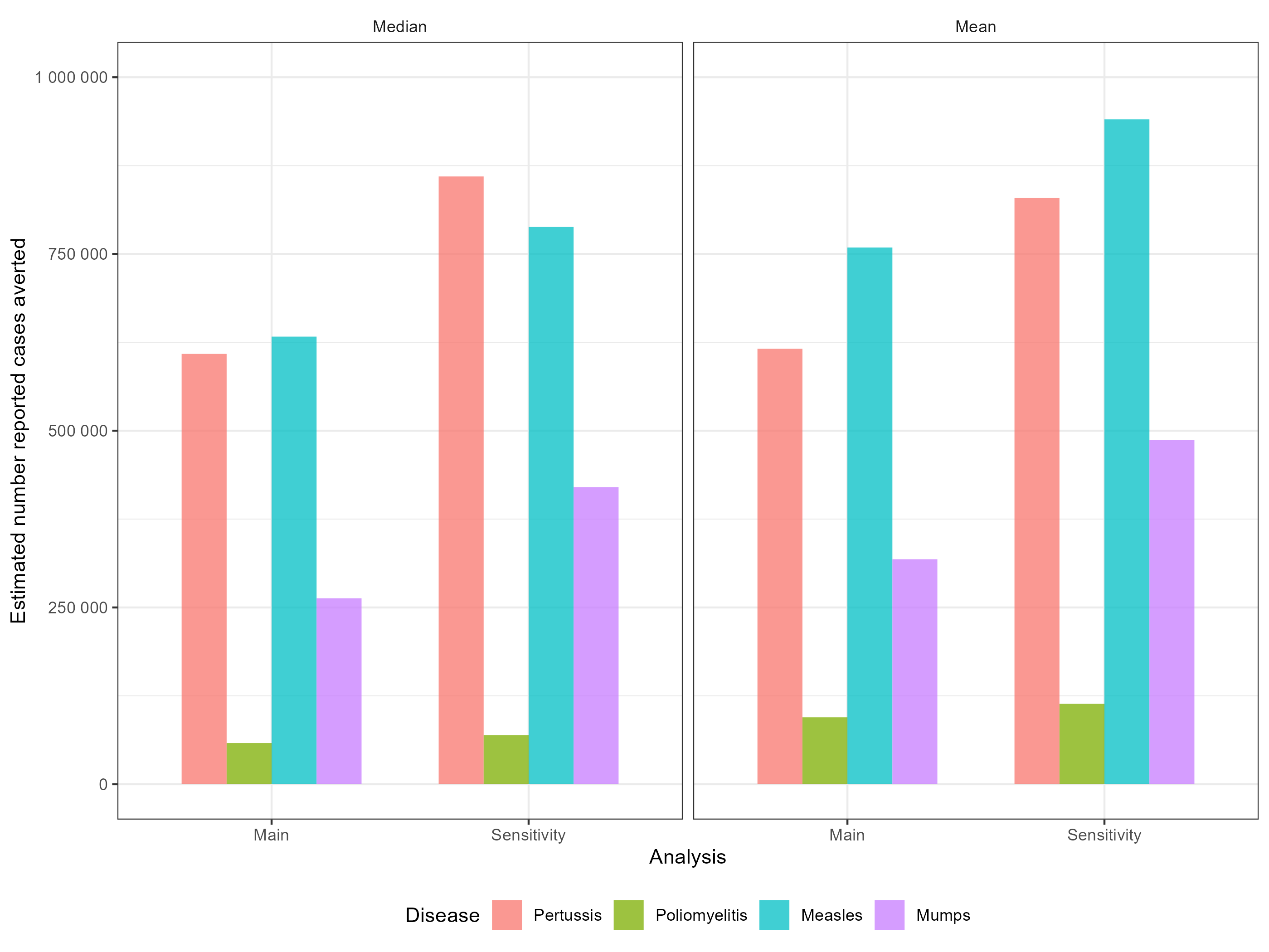


**Figure S2.** Estimated number of reported cases averted using different measures of central tendency (median and mean) and different pre-vaccination periods (main vs. sensitivity analysis).

Note: Main analysis uses all pre-vaccination data, sensitivity analysis uses post-1945 pre-vaccination data.
